# Flow Cytometric Analysis of Efflux by Dye Accumulation

**DOI:** 10.3389/fmicb.2019.02319

**Published:** 2019-10-04

**Authors:** Emily E. Whittle, Simon W. Legood, Ilyas Alav, Punyawee Dulyayangkul, Tim W. Overton, Jessica M. A. Blair

**Affiliations:** ^1^College of Medical and Dental Sciences, Institute of Microbiology and Infection, University of Birmingham, Birmingham, United Kingdom; ^2^School of Cellular and Molecular Medicine, University of Bristol, Bristol, United Kingdom; ^3^School of Chemical Engineering, University of Birmingham, Birmingham, United Kingdom

**Keywords:** flow cytometry, accumulation, efflux, ethidium bromide, gram-negative

## Abstract

Gram-negative infections are increasingly difficult to treat because of their impermeable outer membranes (OM) and efflux pumps which maintain a low intracellular accumulation of antibiotics within cells. Historically, measurement of accumulation of drugs or dyes within Gram-negative cells has concentrated on analyzing whole bacterial populations. Here, we have developed a method to measure the intracellular accumulation of ethidium bromide, a fluorescent DNA intercalating dye, in single cells using flow cytometry. Bacterial cells were stained with SYTO^TM^ 84 to easily separate cells from background cell debris. Ethidium bromide fluorescence was then measured within the SYTO^TM^ 84 positive population to measure accumulation. In *S.* Typhimurium SL1344, ethidium bromide accumulation was low, however, in a number of efflux mutants, accumulation of ethidium bromide increased more than twofold, comparable to previous whole population analysis of accumulation. We demonstrate simultaneous measurement of ethidium bromide accumulation and GFP allowing quantification of gene expression or other facets of phenotype in single cells. In addition, we show here that this assay can be adapted for use with efflux inhibitors, with both Gram-negative and Gram-positive bacteria, and with other fluorescent substrates with different fluorescence spectra.

## Introduction

Antibiotic resistance in Gram-negative bacteria is an ongoing threat. To treat Gram-negative bacterial infections, most antibiotics need to accumulate at high concentrations inside cells in order to reach their targets and be effective. Difficulties arise in obtaining high intracellular levels of antibiotics due to the outer membrane of the Gram-negative bacteria which prevents the access of large and hydrophobic antibiotics into the cell (Nikaido, 1989). Antibiotics that are able to enter through the outer membrane of Gram-negative bacteria can be removed by efflux pumps. Resistance-Nodulation-Division (RND) pumps can extrude a number of antibiotic classes, detergents, and biocides allowing for intrinsic multi-drug resistance (Blair et al., 2015) and are commonly overexpressed in clinical isolates due to mutation (Piddock, 2006; Blair et al., 2015). The outer membrane and efflux systems of Gram-negative bacteria work synergistically to prevent high level accumulation of antibiotics within cells (Krishnamoorthy et al., 2017).

In the literature, there are number of methods used to measure the accumulation of antibiotics and dyes within cells (Blair and Piddock, 2016). These methods are commonly used for assessing accumulation in strains with efflux pump components removed. When measuring accumulation of molecules known to be substrates of efflux pumps, this can provide an indirect assessment of the level of efflux.

Many methods utilize molecules or dyes that are differentially fluorescent when inside and outside cells. In such assays, the fluorescence of cells is measured in a fluorimeter prior to addition of dye and then after dye is added, the increase in fluorescence can be measured as it accumulates within the cell (Coldham et al., 2010; Blair and Piddock, 2016). Examples include DNA intercalating dyes such as ethidium bromide (Olmsted and Kearns, 1977) and Hoechst 33342 (Richmond et al., 2013), which fluoresce when bound to DNA, and nile red and 1,2-dinapthylamine (1,2-DNA) which fluoresce when bound to lipids. These differentially fluorescent compounds are also used for the direct measurement of efflux. The reduction in fluorescence correlated to the removal of dye via efflux pumps. Ethidium bromide is commonly used but nile red (Bohnert et al., 2010) and 1,2-DNA (Bohnert et al., 2011) methods have also been described. An instrument free agar method using ethidium bromide, the cartwheel method, also allows large scale analysis of efflux capacity of strains able to expel the fluorescent dye (Martins et al., 2013).

Accumulation of drugs or other molecules whose fluorescence is not altered by cellular localisation can also be measured although different methods are required. Examples include clinically relevant drugs such as ciprofloxacin (and other naturally fluorescing fluoroquinolones) whose fluorescence is the same inside and outside the cells (Piddock et al., 1999; Blair and Piddock, 2016). To measure accumulation of quinolones, cultures are grown and then incubated with the drug. Cells are washed and lysed, cell debris is removed and the concentration of fluorescent quinolones in the supernatant is measured (Asuquo and Piddock, 1993).

The methods described only measure the ability of a bacteria to accumulate molecules at the bacterial population level. However, the phenotype of a bacterial population is not homogeneous and therefore cells within a population may accumulate different levels of dye/antibiotics. As understanding of heterogeneity and differential gene expression within a bacterial population has increased, it has become desirable to measure the accumulation of molecules at a single cell level. Assessing heterogeneity is important because sub-populations that may accumulate more or less antibiotics than others within a wider population may drive the selection of antimicrobial resistance. Phenotypic variation within a population with regards to efflux may be explained by AcrB bias partitioning between mother and daughter cells during cell division (Bergmiller et al., 2017).

There are a few examples in the literature where fluorescent dyes have been used to measure accumulation in single cells by flow cytometry in certain bacterial species (Sanchez-Romero and Casadesus, 2014; Blair and Piddock, 2016; Hassan et al., 2016; Haynes et al., 2018). Ethidium bromide has been used to measure accumulation in *Salmonella enterica* (Sanchez-Romero and Casadesus, 2014) and *Acinetobacter baumanii* (Hassan et al., 2016). Fluorescein diacetate (FDA) is one of a number of substrates tested in the development of a dye retention assay (Haynes et al., 2018) but nile red and rhodamine 6G have also been used to measure accumulation in the yeast species, *Saccharomyces cerevisiae* expressing *Candida albicans* efflux pumps (Ivnitski-Steele et al., 2009). Single cell analysis has also been described using a femtoliter droplet array which uses the fluorescent dye fluorescein-di-β-galactopyranoside to assess efflux, as well as being used for the analysis of gene expression (Iino et al., 2012, 2018). The natural fluorescence of fluoroquinolones, in this case fleroxacin and ciprofloxacin, has been harnessed to also measure intracellular accumulation within single cells using deep ultraviolet microscopy with a synchrotron beamline (Kascakova et al., 2012). The methods described for both whole population and single cell analysis of efflux vary with regards to difficulty and accessibility as well as the level of analysis of efflux they provide.

Here, we have developed a simple assay that can be used to measure ethidium bromide accumulation in single cells of a number of Gram-negative organisms and in the Gram positive species *Staphylococcus aureus*. We demonstrate that this method is useful for assessing the difference in accumulation between efflux proficient and efflux deficient strains, including comparisons with efflux knockout strains and in the presence of efflux inhibitors. We have also adapted this method to measure fluorescent dyes other than ethidium bromide that have previously been described in literature such as nile red and rhodamine 6G.

## Materials and Equipment

### Strains

Unless otherwise stated, all experiments use *S. enterica* Serovar Typhimurium (hereafter named *S.* Typhimurium (Brenner et al., 2000). SL1344 and isogenic mutants thereof that have been previously published. Details of all strains are shown in Supplementary Table S1. The construction of *Klebsiella pneumoniae* ecl8 *acrB*::Gm is described in the Supplementary Material.

### Media, Buffers and Chemicals

Luria Bertani (Lennox) broth (Sigma Aldrich) was used as the media to grow cultures. 5x Hepes buffered Saline (Alfa Aesar) was diluted to 1x and filter sterilized then used as a buffer for flow cytometry samples. Indole ≥ 99%(Alfa Aesar) was made to a 100 mM stock in 70% ethanol. Carbonyl cyanide 3-chlorophenylhydrazone (CCCP) (Acros Organics) was made to a 10 mM stock in 80% DMSO. Phenylalanine-Arginine β-Naphthylamide (PaβN) (Sigma-Aldrich) was made to a 1000 μg/ml in H_2_O.

### Dyes

Information about all dyes used including providers, excitation/emission maxima, laser line, emission filter and channels used (for the Attune NxT Flow cytometer), and references are provided in Supplementary Table S2 of Supplementary Material. SYTO^TM^84 and SYTO^TM^9 were both made to a stock concentration of 500 μM in water. A 10 mM stock of both ethidium bromide (EtBr) and rhodamine 6G was made up in water. Nile red was made to a 2.5 mM stock in 70% methanol.

### Flow Cytometer and Software

All samples were analyzed using the Attune NxT Flow cytometer and its software package.

## Materials and Methods

### Measuring Ethidium Bromide Accumulation in Gram-Negative Bacteria

Unless otherwise stated, all cultures were incubated in Luria-Bertani broth for 1 h at 37°C, from an overnight culture (4% sub-culture inoculum from overnight culture). Cells were harvested from 300 μL of culture by centrifugation and resuspended in 100 μL 1 × HEPES buffered Saline (HBS) to give a cell count of ∼1 × 10^6^. HBS was used as SYTO dyes are incompatible with phosphate containing buffers. SYTO^TM^ 84 was added to 500 μL of 1 × HBS for a final concentation of 10 μM and ethidium bromide was added to the same sample to give a final concentration of 100 μM. Then 100 μL of bacterial cell suspension was added and incubated for 10 min at room temperature. Cells were analyzed by flow cytometry (Attune Nxt flow cytometer, Life Technologies).

**FIGURE 1:**
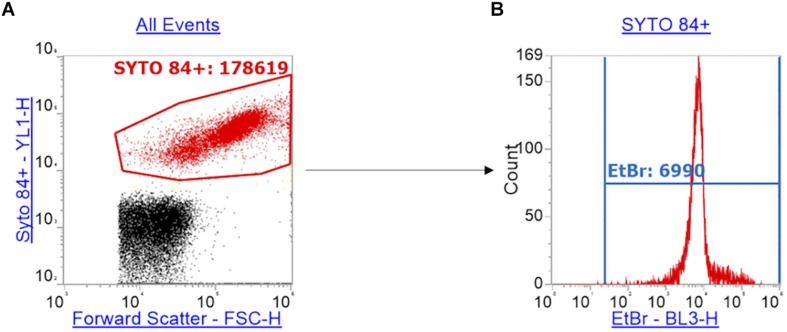
Gating strategy for the measurement of ethidium bromide RFU. Panel **(A)** shows how cells (red) were separated from background debris (black) by gating SYTO^TM^ 84^+^ cells (red) using a plot of the orange fluorescence emission channel YL1-H (SYTO^TM^ 84 fluorescence) against forward scatter (FSC-H). **(B)** Using the red gate of SYTO^TM^ 84^+^ population, a histogram of ethidium bromide fluorescence per cell was plotted using the emission channel BL3-H. The ethidium bromide gate (blue) gives the x-median of ethidium bromide fluorescence to measure relative accumulation.

The emission of SYTO^TM^ 84 was collected in the YL1-H channel. The data was visualized on a plot vs. forward scatter (FSC-H). The SYTO^TM^ 84 dye is used to separate cells from background including cell debris (Figure 1A) and a gate was drawn around the SYTO^TM^ 84^+^ population to separate out cells (Figure 1A: SYTO^TM^ 84^+^ is red). 10,000 events were collected in the SYTO^TM^ 84^+^ gate. The gated SYTO^TM^ 84^+^ cells were then analyzed for ethidium bromide fluorescence from the BL3-H channel (Figure 1B: EtBr RFU from blue gate). The values that were taken for relative fluorescence units (RFU) is the X-median, where all distibutions were based on a single population. The optimized PMT voltages used were as follows: FSC at 700, SSC at 500, YL1 at 500 and BL3 at 400.

### Flow Cytometry Set Up and Gating/Compensation Using Ethidium Bromide

The excitation of SYTO^TM^ 84 and ethidium bromide are at different wavelengths and therefore there was limited spillover of the emission spectra of these two fluorophores. However, compensation was still set for the YL1-H and BL3-H parameters against and unstained control using the Attune NxT compensation setup.

### Measurement of Rhodamine 6G Accumulation

The excitation wavelength used for Rhodamine 6G was 488 nm, and so the methods used to measure this dye are the same as measuring accumulation of ethidium bromide.

### Measurement of Nile Red Accumulation

As above, samples were set up exactly the same apart from the addition of nile red (final concentration of 75 μM) in place of ethidium bromide. Optimisation for concentrations is not shown.

Nile red has an excitation of 549 nm and emission of 628 nm in the presence of phospholipids, and in a neutral lipid environment (tryglycerides), the fluorescence shifts to ex/em of 510/580 nm (Greenspan and Fowler, 1985). For this reason, SYTO^TM^ 9 was used in place of SYTO^TM^ 84. Nile red ex/em overlaps with SYTO^TM^ 84 in both phospholipid and tryglyceride staining. All other sample preparation was the same as with ethidium bromide. The SYTO^TM^ 9 emission channel used was BL2-H for green fluorescence and SYTO^TM^ 9^+^ cells were gated so as to separate from cell debris. The SYTO 9^+^ population was then used to measure nile red fluorescence using the YL1-H channel for orange fluorescence. Compensation was set but as with ethidium bromide, there should be no spillover between SYTO^TM^ 9 and nile red emissions.

### Measuring Accumulation in the Presence of Proton Uncoupler, CCCP and RND Pump Inhibitor, PAβN

To analyse the effect of CCCP on dye accumulation in *S.* Typhimurium, a sub-inhibitory concentration (100 μM) was added to 500 μL of 1 × HBS, followed by ethidium bromide and SYTO^TM^ 84, and SL1344 as above. 100 μM of CCCP was used based on previous direct efflux assays for Gram-negative bacteria (Smith and Blair, 2014). To analyse the effect of CCCP on dye accumulation in *S. aureus*, 10 μM was added to 500 μL of 1 × HBS, followed by ethidium bromide and SYTO^TM^ 84. Samples could then be analyzed for accumulation after 10 min of incubation. The CCCP concentration for inhibition of pumps in *S. aureus* was decided based on analysis of the following concentrations: 1 μM, 10 μM, 50 μM and 100 μM and the higher concentrations affected the SYTO^TM^ 84+ population possibly due to cell death. Optimisation for the concentration is not shown.

To analyse the effect of PAβN on nile red accumulation in *S.* Typhimurium, a concentration of 50 μg/ml was added to 500 μL of 1 × HBS, followed by nile red and SYTO^TM^ 9, and SL1344 as above. We were unable to use ethidium bromide in the presence of PaβN because there was no difference in accumulation in the presence or absence of the RND inhibitor and previous studies suggest this (Lomovskaya et al., 2001; Kern et al., 2006; Viveiros et al., 2008; Machado et al., 2017).

### Flow Cytometry Set Up Using Ethidium Bromide and GFP

In order to show that measurements of ethidium bromide accumulation can be combined with measurements of GFP, we used a transcriptional reporter plasmid encoding a *ramA* promoter upstream of a *gfp* reporter gene. *ramA* is a transcriptional activator of the *acrAB* operon, therefore upregulating the AcrAB-TolC efflux pump in response to signals such as indole (Nikaido et al., 2008). When using strains containing GFP transcriptional reporter plasmids, cultures were supplemented with 50 μg/mL of ampicillin and were grown for 2 h (OD_600_ = 0.6) to correspond to published data reporting increased *ramA* transcription (Lawler et al., 2013). After 2 h, 200 μL samples of the culture were taken, and to those which were to be induced, a final concentration of 2 mM of indole was added to the culture. This was then incubated for 30 min. Cells were then resuspended in 1x HBS and samples were set up as previously described.

**FIGURE 2:**
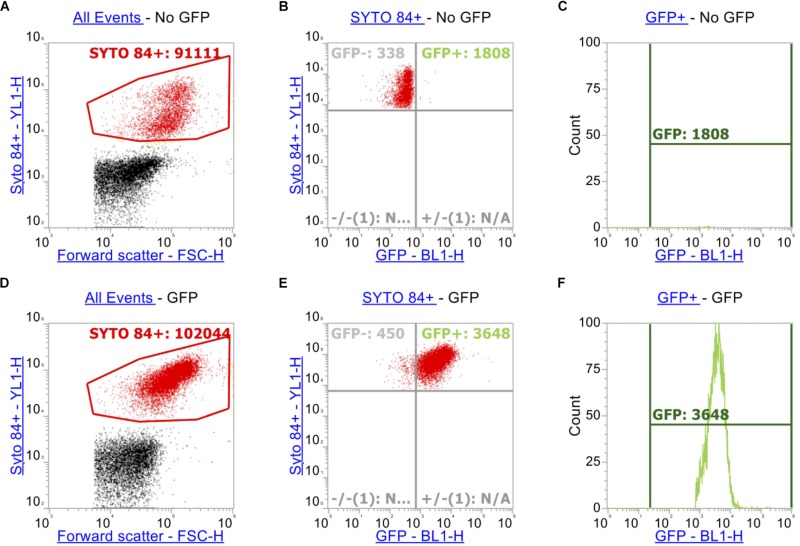
Gating strategy when measuring GFP alongside ethidium bromide. As above, SYTO^TM^ 84^+^ cells were gated above in red **(A,D)**. Panels **(A–C)** represents strain SL1344 expressing no GFP. **(B,E)** Plots YL1-H for SYTO^TM^ 84 against BL1-H for GFP. Only SYTO^TM^ 84^+^ cells are analyzed in these plots and the GFP^+^ gate (Blue, right hand side) represents cells that are expressing GFP only and excluding cells fluorescing in BL1-H channel by spillover **(C,F)**.

In order to measure accumulation of ethidium bromide in addition to measuring GFP (in this case a transcriptional reporter of *ramA*), additional gating and compensation was required. In addition to the gating strategy described above for SYTO^TM^ 84 and ethidium bromide (Figures 2A,D), a plot showing YL1-H (SYTO^TM^ 84) vs. BL1-H (for GFP) of SYTO^TM^ 84^+^ cells was set up (Figures 2B,E). A quadrant gate was then added to this dot plot to specify cells that expressed GFP. The GFP^+^ population (blue) was then analyzed by a histogram plot (Figures 2C,F) to calculate an X-median RFU for fluorescence of GFP. GFP and ethidium bromide have similar excitation and emission maxima therefore compensation was required to adjust for spillover of emission of ethidium bromide and GFP in both channels.

### Statistical Analysis

A one-way ANOVA and Dunnett’s multiple comparison test were used on Prism Graphpad to assess significance and *P*-values of SL1344 compared to Δ*acrB*, Δ*tolC*, Δ*acrAE*, and Δ4PAP (Δ*acrA*, Δ*acrE*, Δ*mdtA*, Δ*mdsA)* strains. Unpaired *T*-tests were run of each wild-type strain of *E. coli, K. pneumonia*, and *Pseudomonas aeruginosa* against their efflux deficient strain. Unpaired *T*-tests were also used for nile red, rhodamine 6G and GFP and also in the presence of inhibitor, CCCP. All *P*-values and related asterisks are shown in Supplementary Table S3.

## Results

### Assay Development

**FIGURE 3:**
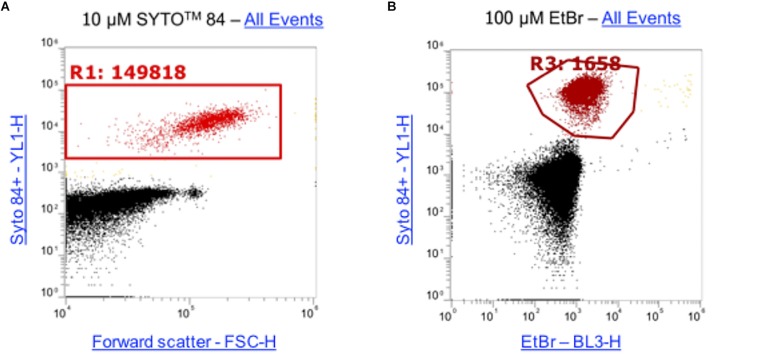
Concentrations of SYTO^TM^ 84 and ethidium bromide used in this assay. **(A)** 10 μM SYTO^TM^ 84 was used and a population gated (R1) on a orange fluorescence (YL1-H) versus forward scatter (FSC-H) dot plot to identify SYTO^TM^ 84 fluorescence. The concentration was used as it gave the best separation of cells (red) from background cell debris and other particulate noise (black). **(B)** 100 μM was the optimum concentration of ethidium bromide used as shown via a dot plot of YL1-H (SYTO^TM^ 84) vs BL3-H (ethidium bromide). Both plots show all events.

Ethidium bromide only fluoresces when bound to DNA therefore the level of fluorescence corresponds to the level of intracellular accumulation. This makes it a powerful tool for analyzing the effects of accumulation in strains that lack efflux pumps. Traditionally, accumulation of substrates such as ethidium bromide has been measured on the level of a whole bacterial population but the method presented here allows accumulation to be measured in single cells within a large population. To optimize this assay to use for flow cytometry, cultures were incubated for 1 h. The DNA intercalating dye SYTO^TM^ 84 was used to distinguish cells from background particulate noise (e.g., cell debris, medium components) and was selected as its fluorescence emission did not overlap the emission of ethidium bromide. A range of concentrations of SYTO^TM^ 84 were tested (0.1 μM, 0.5 μM, 1 μM, 5 μM, 10 μM) but 10 μM was the optimum concentration for use by flow cytometry because there was better separation between cells and debris and a distinct population was visualized (Supplementary Figure S3). To optimize the concentration of ethidium bromide used in the assays, 5 concentrations were tested (10 μM, 25 μM, 50 μM, 75 μM, 100 μM). A 100 μM concentration of ethidium bromide gave the highest mean fluorescence in *S.* Typhimurium wild type strain SL1344 stained with SYTO^TM^ 84 and the greatest difference between a wild type and Δ*acrB.* Example plots using the final concentrations for both SYTO^TM^ 84 and ethidium bromide are shown (Figure 3), data from other concentrations can be found in Supplementary Figure S3.

### Accumulation of Ethidium Bromide Can Be Measured in *Salmonella* Efflux Mutants

Using the optimized assay, accumulation of ethidium bromide was measured in wild type *Salmonella* SL1344 and the isogenic efflux mutants Δ*acrB*, Δ*tolC*, Δ*acrAE*, and a Δ4PAP strain lacking the four periplasmic adaptor protein (PAP) genes (Δ*acrA*, Δ*acrE*, Δ*mdtA*, Δ*mdsA).*

**FIGURE 4:**
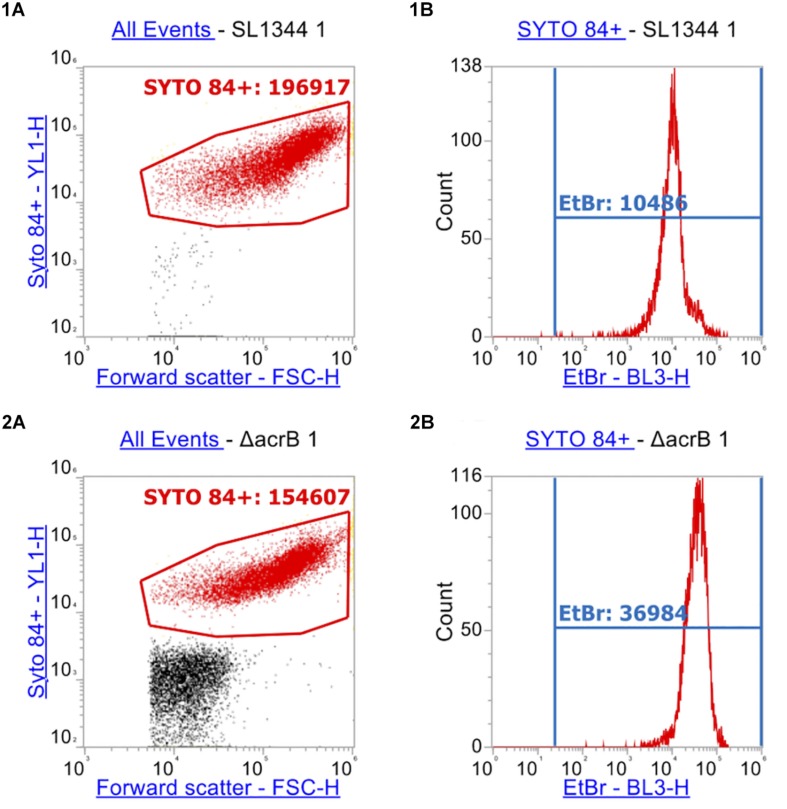
Flow cytometry plots of SL1344 and SL1344 Δ*acrB*. (1) shows plots of SL1344 and (2) shows plots of efflux mutant SL1344 Δ*acrB.* **(A)** Separation of cells from background cell debris using channels YL1-H vs. FSC-H. SYTO 84^+^ cells are gated (red gate) with the X-median value given. 10,000 events were collected inside the SYTO 84^+^ gate. From this, the red population is analyzed in **(B)** plots where the BL3-H channel was used to measure the X-median of ethidium bromide fluorescence in a new gate (EtBr in Blue). Fluorescence of ethidium bromide was only measured from the SYTO 84^+^ population.

**FIGURE 5:**
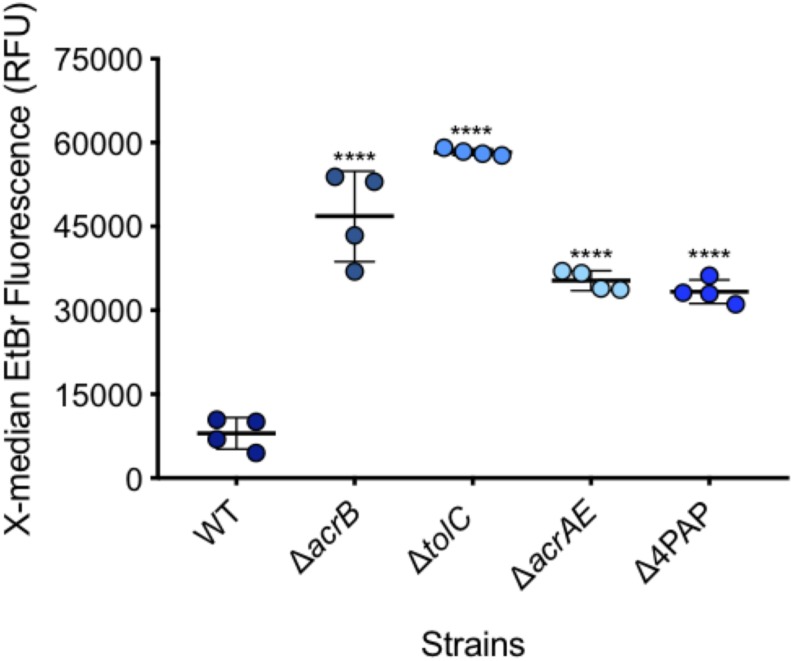
Accumulation of ethidium bromide in WT SL1344 and efflux deficient strains. Single circles represent the X-median value of ethidium bromide fluorescence from 10,000 cells within a biological replicate. 4 biological replicates for each strain are shown, with a long bar to show the mean and standard error of the mean (SEM) error bars. WT had a mean RFU of 8024. This is significantly lower than the mean RFUs of Δ*acrB* (46824 RFU), Δ*tolC* (58321 RFU), Δ*acrAE* (35314 RFU) and D4PAP (33359 RFU). Significance values were based on a one-way ANOVA and Dunnett’s multiple comparison test of all strains compared to WT. All *P*-values were < 0.0001 which is represented by ^****^ above strain plots in this figure.

After measuring 10,000 cells, the X-median of ethidium bromide fluorescence was taken in the distinct population of SYTO^TM^ 84^+^ cells (Figures 4, 5). Low level fluorescence could be detected in the WT SL1344 with a mean value of 8024 RFU. Fluorescence was sixfold higher in the absence of AcrB, sevenfold higher when the outer membrane channel (TolC) was removed and fourfold higher in both Δ*acrAE* and Δ4PAP strains. These data are in agreement with previous whole population data showing that efflux deficient strains accumulate more ethidium bromide (Coldham et al., 2010; Blair and Piddock, 2016).

### Other Substrates

Ethidium bromide is commonly used in assays for direct efflux measurement but this assay has also been adapted to use the lipophilic dye, nile red (Bohnert et al., 2010; Bohnert et al., 2011; Blair and Piddock, 2016) and Rhodamine 6G (Ivnitski-Steele et al., 2009). Nile red fluoreces when in the presence of both phospholipids and tryglycerides with different excitation and emission. Here, we also show that the flow cytometry assay described can be adapted for use with these structurally diverse efflux substrates.

**FIGURE 6:**
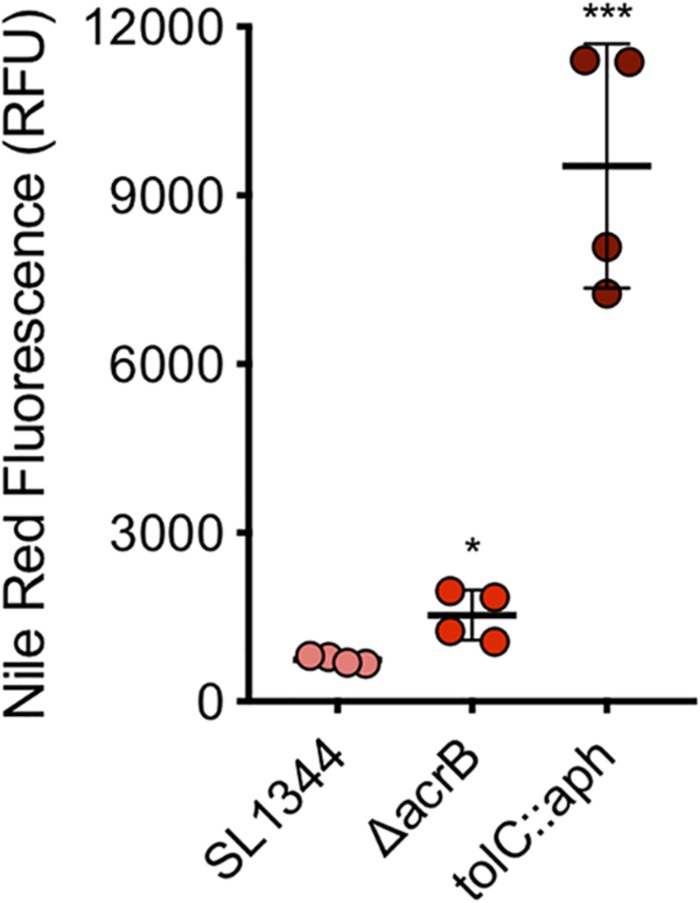
Accumulation of nile red in WT, Δ*acrB* and *tolC::aph* in *S.* Typhimurium. Single circles represent the X-median value of nile red fluorescence from 10,000 cells within a biological replicate using WT (pink), Δ*acrB* (red) and Δ*tolC* (dark red). 4 biological replicates for each strain are shown, with a long bar to show the mean and SEM error bars. Significance values were based on an unpaired *T*-test comparing the efflux deficient strain to WT. ^∗^ and ^∗∗∗^ correspond to *P*-values shown in Supplementary Table S3.

A series of concentrations of each dye were tested to determine the optimal concentration. A final sub-inhibitory concentration of 75 μM of nile red was used. The average fluorescence of nile red in SL1344 was 744 RFU while in the absence of AcrB the average fluorescence was twofold higher (1535 RFU, *P*-value < 0.05) (Figure 6). However, in the absence of TolC, the outer membrane component of the efflux pump, accumulation of nile red was 13-fold higher than SL1344 (9458 RFU, *P*-value 0.0002).

Rhodamine 6G was also tested (Supplementary Figure S4) and accumulation could be measured, however, accumulation between a WT and efflux mutants did not significantly differ. Rhodamine 6G has been reported as a substrate of the AcrAB-TolC pump in *S.* Typhimurium (Horiyama et al., 2010), however, further optimization such as longer incubation for differences in accumulation may be required.

### Efflux Inhibitors

This assay can also be applied to measure the effect of efflux inhibitors. The accumulation of ethidium bromide was measured in WT SL1344 in the presence of the efflux dissapator Carbonyl Cyanide m-Chlorophenylhydrazine (CCCP). CCCP is a proton motive force inhibitor therefore dissipating the energy source of efflux pumps that require the proton motive force (Mahamoud et al., 2007). The RND pump inhibitor PaβN was used to measure the accumulation in nile red in WT SL1344. Previous studies show that PaβN does not significantly change the MIC or accumulation of ethidium bromide in Gram-negative bacteria (Lomovskaya et al., 2001; Kern et al., 2006; Viveiros et al., 2008; Machado et al., 2017).

**FIGURE 7:**
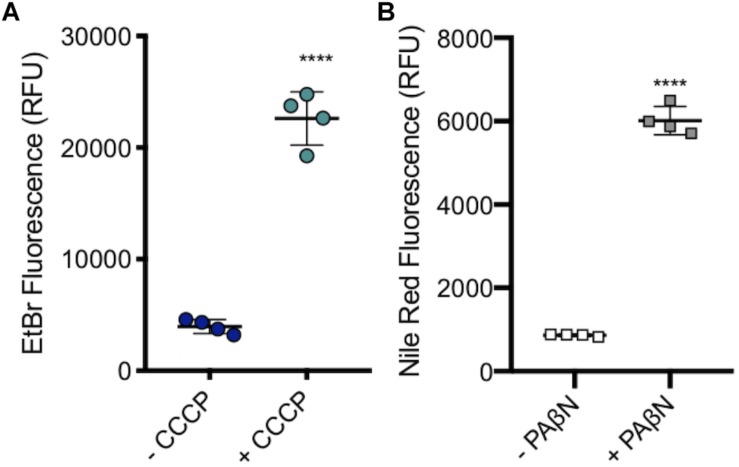
Accumulation of ethidium bromide in SL1344 in the absence and presence of 100 μM CCCP and 50 μg/ml of PAβN. **(A)** Single dots represent the X-median value of ethidium bromide fluorescence from 10,000 cells within a biological replicate. 4 biological replicates for each condition are shown, with a long bar to show the mean and standard error of the mean (SEM) error bars. WT had a mean RFU of 3936 (blue); +CCCP (green) the RFU increased sixfold to 22596 RFU. **(B)** Single squares represent the X-median value of nile red fluorescence in the presence and absence of PAβN, from 10,000 cells within a biological replicate. 4 biological replicates for each condition are shown, with a long bar to show the mean and SEM error bars. WT had a mean RFU of 863 (white); + PaβN (gray) RFU showed a sevenfold increase. For both **(A)** and **(B)** Significance values were based on an unpaired *T*-test. The *P*-values were < 0.0001 which was represented by ^****^ in this figure.

A subinhibitory concentration (100 μM) of CCCP was added to SL1344 to measure the level of accumulation when efflux pumps are inhibited, without killing the cells. As with efflux pump gene deletion, there was a significant increase in the level of ethidium bromide accumulation upon the addition of CCCP (Figure 7A).

PaβN, at 50 μg/ml, was added to SL1344 to measure the level of nile red accumulation in the absence of RND pump function, without killing the bacterial cells. Experimental data showed that there was a significant increase in the accumulation of nile red in SL1344 cells that were treated with PaβN, a similar result to the addition of CCCP and efflux gene deleted strains. This shows that this method could be used to screen for putative efflux inhibitors (Figure 7B).

### Parallel Measurement of Ethidium Bromide Accumulation and GFP

To answer complex biological questions it is often desirable to measure two phenotypic characteristics in the same sample in parallel and doing this in single cells can be particularly powerful. The ethidium bromide accumulation assay described can be combined with measurement of green fluorescence, and therefore could be adapted to measure alongside fluorophores such as from GFP or a FITC labeled antibody, to allow this complexity. Excitation of both ethidium bromide and GFP was at 488 nm but their emission walevelengths differ so both can be analyzed simultaneously.

**FIGURE 8:**
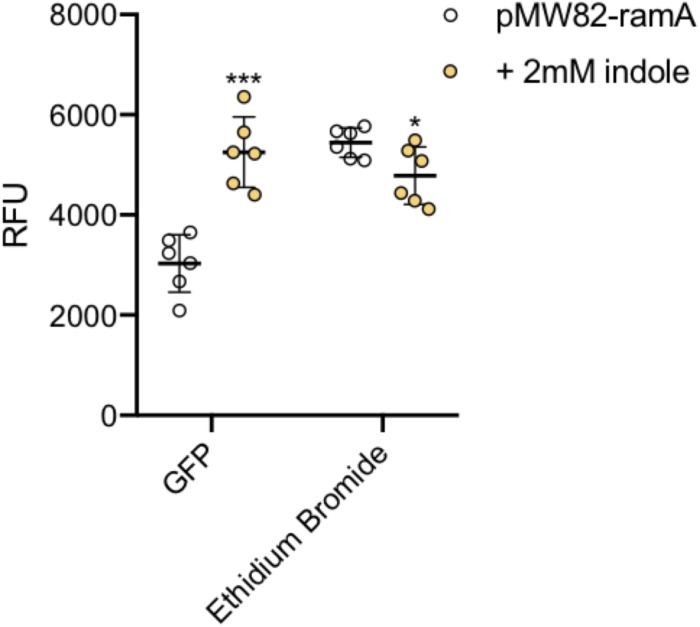
Accumulation of ethidium bromide and fluorescence of GFP. The ethidium bromide and GFP fluorescence (*x*-axis) were measured in SL1344 pMW82-ramA in the absence (white) and presence (orange) of 2 mM indole. 6 biological replicates for each strain are shown, with a long bar to show the mean and standard error of the mean (SEM) error bars. Ethidium bromide fluorescence of the strains is shown and is consistent with results above. The X-median RFU for GFP is shown without (white) and with (orange) the addition of indole in strains containing a pMW82-*ramA* transcriptional reporter. Without indole, the mean RFU is 2963 but with indole it is 5230 RFU. Unpaired *T*-tests were run to compare fluorescence of GFP and ethidium bromide with and without indole. The *P*-value for GFP (^∗∗∗^) was 0.0001; The *P*-value for EtBr (^∗^) was 0.0315.

To illustrate this we have used the wild-type *S.* Typhimurium SL1344 transformed with a transcriptional reporter plasmid encoding the promoter of the *ramA* gene fused to *gfp* (pMW82-*ramA*) to show it is possible to monitor dye accumulation and gene expression in parallel in single cells (Figure 8). RamA is a positive regulator of the efflux pump genes *acrAB*. GFP fluorescence corresponding to *ramA* transcription and ethidium bromide accumulation were measured in the same cells, in the absence and presence of indole, a known inducer of *ramA* (Nikaido et al., 2008; Lawler et al., 2013). Upon addition of indole there was a twofold increase in GFP fluorescence showing increased transcription of *ramA*, and this was associated with significantly lower accumulation of ethidium bromide per cell within the same population, presumably through induction of the AcrAB efflux pump.

### Ethidium Bromide Accumulation Can Be Measured in Both Gram-Negative and Gram-Positive Bacteria

**FIGURE 9:**
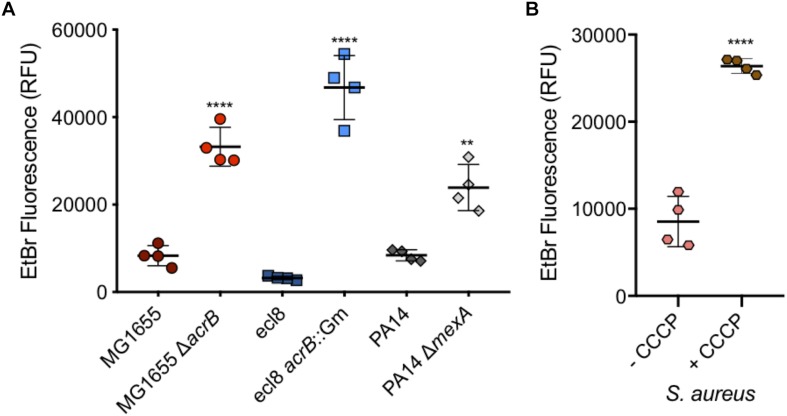
Accumulation of ethidium bromide in WT and efflux mutants of *Escherichia coli*, *Klebsiella pneumonia*, and *P. aeruginosa*, and in *Staphylococcus aureus* with CCCP. **(A)** *E. coli* MG1655 (dark red circles) had an average fluorescence of 8310 RFU and was significantly lower (fourfold) than its Δ*acrB* (red circles) equivalent at 33233 RFU. *Klebsiella pneumoniae* ecl8 (dark blue squares) had an average RFU of 3225, 14-fold lower than its *acrB*::Gm inactivated strain with a RFU of 46764. *P. aeruginosa* strain PA14 (gray diamonds) had ethidium bromide fluorescence of 8401 RFU which was significantly lower than the fluorescence for its Δ*mexA* strain at RFU (threefold difference). **(B)** *S. aureus* ethidium bromide accumulation was measured in the absence (pink) and presence (brown) of CCCP. In the presence of CCCP, accumulation was threefold higher (*P* < 0.0001). 4 biological replicates for each strain are shown, with a long bar to show the mean and standard error of the mean (SEM) error bars. Statistical tests were unpaired *T*-tests between individual WT and efflux deficient strain. Results were considered significantly different if *p*-value ≤ 0.05. ^∗∗^ and ^****^ correspond to *P*-values shown in Supplementary Table S3.

*Salmonella* Typhimurium is one of many Gram-negative organisms that are of concern with regards to antimicrobial resistance and where efflux has been shown to contribute to multi-drug resistance in clinical isolates. For this reason, and to confirm the use of this assay in measuring accumulation in other species, WT and efflux mutants of *Escherichia coli*, *K. pneumoniae* and *P. aeruginosa* strains were also tested. For each of the three Gram-negative species tested, a significant increase in accumulation could be seen in the absence of efflux (Figure 9A). This shows that this assay can be used with many species of Gram-negative bacteria to test substrate accumulation.

Ethidium bromide accumulation was also measured in *S. aureus* when efflux pumps were inhibited by CCCP (Figure 9B). This confirmed that this assay can also be used when measuring accumulation in Gram-positive bacteria.

## Discussion

Traditionally measurement of dye or drug accumulation has focused on measurement of whole bacterial populations and this approach has been critical in the investigation of efflux pumps and assessing potential efflux inhibitors. It is now evident that populations of bacteria are not homogeneous in phenotype, and that stochastic gene expression leads to a heterogeneous population and studies have shown its importance in measuring accumulation (Sanchez-Romero and Casadesus, 2014; Davis and Isberg, 2016). For this reason, especially when assessing inhibition of efflux, it may be important to analyse accumulation at the single cell level. If a population of cells with inhibited efflux contains a sub-population that accumulates low concentrations of drug, this may lead to acquisition of mutations and therefore resistance. The assay described here allows the measurement of substrate accumulation in single cells and therefore strengthens our understanding of a cell ability to accumulate drug/dye across a population.

The variations of the assay described highlight its versatility. Not only were we able to show the accumulation of ethidium bromide in *S. enterica* Serovar Typhimurium, but also its use in other Gram-negative species such as *E. coli* (Bohnert et al., 2008), *K. pneumoniae* (Srinivasan et al., 2014) and *P. aeruginosa* (Mao et al., 2002; Stoitsova et al., 2008) and the Gram-positive organism, *S. aureus.* The *P. aeruginosa mexA* mutant only showed a slight (but significant) increase in ethidium bromide accumulation as compared to the wild type. This organism has an intrinsically impermeable membrane and a large number of efflux systems (Hancock, 1998; Breidenstein et al., 2011) therefore removing a single efflux gene (*mexA*) may not have as a dramatic effect as in the other organisms tested here. This highlights a potential difficulty when assessing drug accumulation in Gram-negatives such as *P. aeruginosa*. Some species such as *E. coli* have more than one pump that export ethidium bromide. Previous studies showed the application of nile red (Bohnert et al., 2010) in efflux assays and we have shown that our flow cytometry assay can be adapted to work with this substrate. In future, our assay could be adapted to measure accumulation of many other fluorescent subtrates and may therefore also be used to identify if a substrate is exported by a specific efflux pump.

A large number of antimicrobial resistant clinical isolates often have high expression of efflux pumps making it an attractive new target for therapeutics. The use of CCCP, which stops efflux through the proton motive force, shows that this assay has potential applications in efflux inhibitor screening. Finally, coupling our assay to Fluorescence-Activated Cell Sorting (FACS) will permit physical selection of single cells with particular efflux properties, potentially in combination with other phenotypic markers such as gene expression, aiding screening applications.

## Data Availability Statement

The datasets generated for this study are available on request to the corresponding author.

## Author Contributions

JB and EW designed this assay. EW performed all experiments apart from *S. aureus* experiments performed by SL and nile red experiments performed by IA, and analyzed all the data. PD constructed the *Klebsiella pneumoniae* ecl8 mutant strain. EW, JB, and TO wrote the manuscript.

## Conflict of Interest

The authors declare that the research was conducted in the absence of any commercial or financial relationships that could be construed as a potential conflict of interest.
