# Supplementary material for: Flow Cytometric Analysis of Efflux by Dye Accumulation
Source: Front Microbiol. 2019 Oct 4;10:2319. doi: 10.3389/fmicb.2019.02319 (PMC6787898; doi:10.3389/fmicb.2019.02319)
Supplement: Supplementary file 1 [file Table_1.docx]

**Supplementary material**

**1 Materials**

**Table S1 Strain list**

| **Organism** | **Strain** | **Reference:** |
| --- | --- | --- |
| *Salmonella* Typhimurium: | SL1344 | (Wray and Sojka 1978) |
|  | SL1344 ∆*acrB* | (Eaves, Ricci et al. 2004) |
|  | SL1344 ∆*tolC* | (Buckley, Webber et al. 2006) |
|  | SL1344 ∆*acrAE* | (Smith and Blair 2014) |
|  | SL1344 ∆*acrA* ∆*acrE* ∆*mdsA* ∆*mdtA* | McNeil et al. (Submitted) |
|  | SL1344 + pMW82-*ramA* | (Lawler, Ricci et al. 2013) |
| *Escherichia coli*: | MG1655 |  |
|  | MG1655 ∆*acrB* | (Wang-Kan, Blair et al. 2017) |
| *Klebsiella pneumoniae:* | ecl8 |  |
|  | ecl8 *acrB*::Gm |  |
| *Pseudomonas aeruginosa:* | PA14 | (pa14.mgh.harvard.edu , Liberati, Urbach et al. 2006) |
|  | PA14 ∆*mexA* | (pa14.mgh.harvard.edu , Liberati, Urbach et al. 2006) |
| *Staphylococcus aureus* | ATCC 29213 |  |

**Table S2 Fluorescent dyes used in this study**

| **Dye/Fluorescent protein** | **Company** | **Ex/em (nm)** | **Laser** | **Emission filter (nm)** | **Channel name** |
| --- | --- | --- | --- | --- | --- |
| SYTO^TM^ 84 Orange Fluorescent Nucleic Acid Stain | Invitrogen;  Fisher | 567/582 | Yellow  (561 nm) | 585/16 | YL1 |
| SYTO^TM^ 9 Green Fluorescent Nucleic Acid Stain | Invitrogen;  Fisher | 485/498 | Blue  (488 nm) | 590/40 | BL2 |
| Ethidium bromide | 95%, Acros Organics | 482/616 | Blue  (488 nm) | 695/40 | BL3 |
| Nile red | 99%, Acros Organics | 549/628 510/580 | Yellow  (561 nm) | 585/16 | YL1 |
| Rhodamine 6G | Sigma Aldrich | 528/551 | Blue  (488 nm) | 530/30 | BL1 |
| GFP (pMW82-*ramA*) | (Lawler, Ricci et al. 2013) | 488/510 | Blue  (488 nm) | 530/30 | BL1 |

**2. Methods**

**Construction of the *Klebsiella pneumoniae*ecl8 *acrB*::Gm strains**

*Klebsiella pneumoniae* ecl8 *acrB*::Gm was constructed using the pKNOCK-Gm suicide vector methods described previously (Alexeyev 1999). The marker used was gentamicin acetyltransferase (*aacC1*). Briefly, using forward (5’-CAATACGCAAGAGTTTGGCA-3’) and reverse (5’-CACCAGGATGATCGCTTC-3’) oligonucleotides, a 331 bp fragment was inserted into the vector at the *Sma*I site of the pKNOCK-Gm plasmid. The recombinant plasmid was transferred into *K. pneumoniae* ecl8 wild-type cells by conjugation. *K. pneumoniae* ecl8 *acrB*::Gm was selected by gentamicin and insertion was verified by PCR using forward (5’-CCGCAACAGGGTGTTAC-3’) and reverse (5’-GAATCTGTCCCATCGATTTAC-3’) oligonucleotides.

**Table S3 P-values and corresponding asterisks based on statistical analysis**

| **P-value** | **Asterisk** |
| --- | --- |
| >0.05 | ns |
| 0.01 – 0.05 | * |
| 0.001 – 0.01 | ** |
| 0.0001 - 0.001 | *** |
| <0.0001 | **** |

**3 Results**


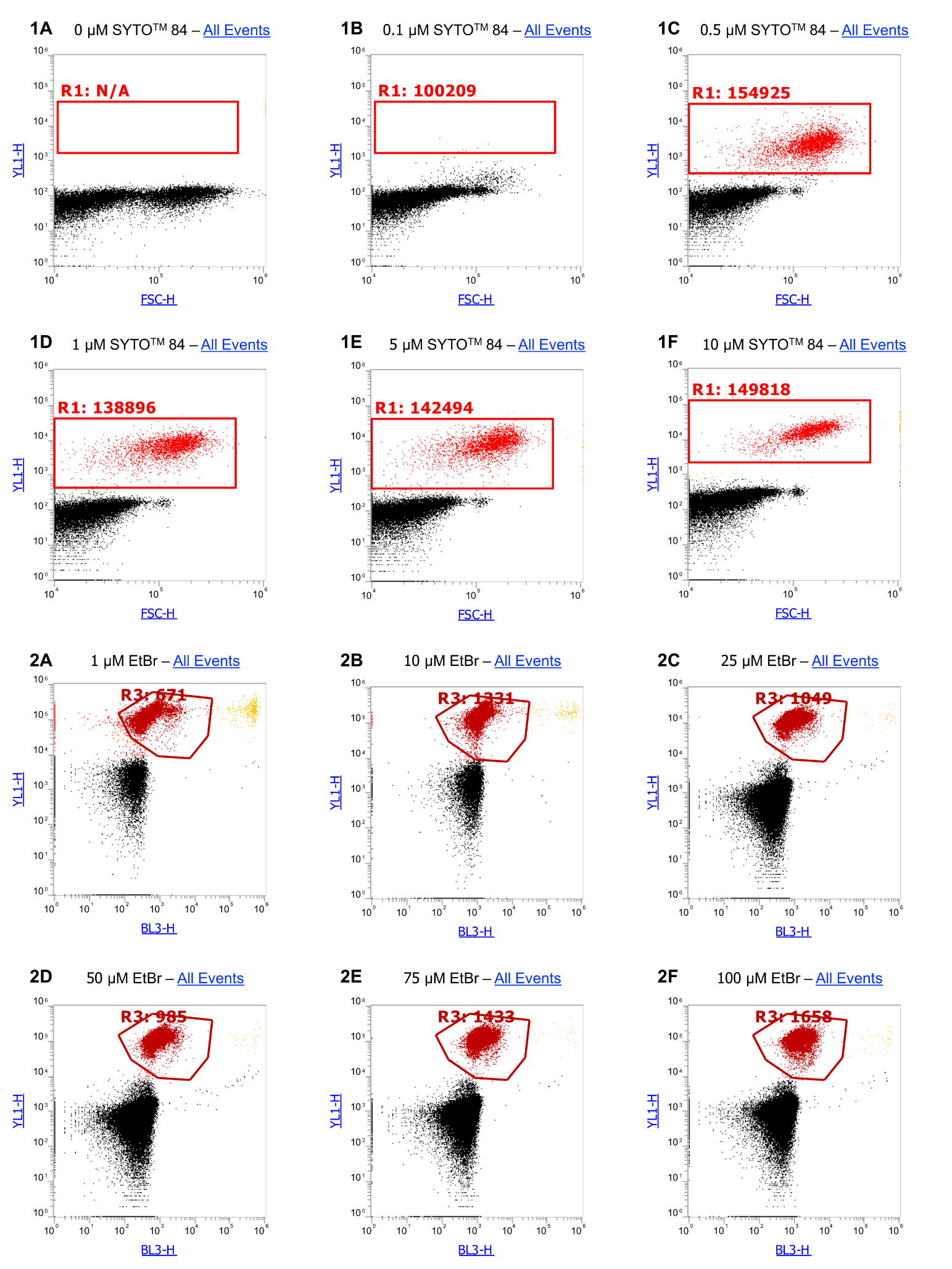


**Figure S3 Optimal concentrations for SYTO^TM^ 84 and Ethidium Bromide.** 1A-1F show increasing concentrations of SYTO^TM^ 84 (0 µM – 10 µM) on dot plots of orange fluorescence (YL1-H) versus forward scatter (FSC-H) . 2A-2F show increasing concentrations of ethidium bromide (10 µM, 25 µM, 50 µM, 75 µM, 100 µM) in the presence of 10 µM SYTO^TM^ 84 on dot plots of orange fluorescence (YL1-H) versus red fluorescence (BL3-H).

**Figure S4 Accumulation of rhodamine 6G in WT and ∆acrB S.Typhimurium.** Single dots represent the X-median value of rhodamine 6G fluorescence from 10,000 cells within a biological replicate using WT (pink) and ∆acrB (purple). The strains were not significantly different based on an unpaired T-test.

**3 References**

Alexeyev, M. F. (1999). "The pKNOCK series of broad-host-range mobilizable suicide vectors for gene knockout and targeted DNA insertion into the chromosome of gram-negative bacteria." Biotechniques **26**(5): 824-826, 828.

Buckley, A. M., M. A. Webber, S. Cooles, L. P. Randall, R. M. La Ragione, M. J. Woodward and L. J. Piddock (2006). "The AcrAB-TolC efflux system of Salmonella enterica serovar Typhimurium plays a role in pathogenesis." Cell Microbiol **8**(5): 847-856.

Eaves, D. J., V. Ricci and L. J. Piddock (2004). "Expression of acrB, acrF, acrD, marA, and soxS in Salmonella enterica serovar Typhimurium: role in multiple antibiotic resistance." Antimicrob Agents Chemother **48**(4): 1145-1150.

Lawler, A. J., V. Ricci, S. J. Busby and L. J. Piddock (2013). "Genetic inactivation of acrAB or inhibition of efflux induces expression of ramA." J Antimicrob Chemother **68**(7): 1551-1557.

Liberati, N. T., J. M. Urbach, S. Miyata, D. G. Lee, E. Drenkard, G. Wu, J. Villanueva, T. Wei and F. M. Ausubel (2006). "An ordered, nonredundant library of Pseudomonas aeruginosa strain PA14 transposon insertion mutants." Proc Natl Acad Sci U S A **103**(8): 2833-2838.

pa14.mgh.harvard.edu. "PA14 Transposon Insertion Mutant Library." from <http://ausubellab.mgh.harvard.edu/cgi-bin/pa14/home.cgi>.

Smith, H. E. and J. M. Blair (2014). "Redundancy in the periplasmic adaptor proteins AcrA and AcrE provides resilience and an ability to export substrates of multidrug efflux." J Antimicrob Chemother **69**(4): 982-987.

Wang-Kan, X., J. M. A. Blair, B. Chirullo, J. Betts, R. M. La Ragione, A. Ivens, V. Ricci, T. J. Opperman and L. J. V. Piddock (2017). "Lack of AcrB Efflux Function Confers Loss of Virulence on Salmonella enterica Serovar Typhimurium." MBio **8**(4).

Wray, C. and W. J. Sojka (1978). "Experimental Salmonella typhimurium infection in calves." Res Vet Sci **25**(2): 139-143.
